# The Function of a Spindle Checkpoint Gene *bub-1* in *C. elegans* Development

**DOI:** 10.1371/journal.pone.0005912

**Published:** 2009-06-15

**Authors:** Xiangming Wang, Min Liu, Weida Li, Christopher D. Suh, Zuoyan Zhu, Yishi Jin, Qichang Fan

**Affiliations:** 1 School of Life Sciences, Peking University, Beijing, China; 2 Department of Molecular, Cell and Developmental Biology, Sinsheimer Laboratories, University of California Santa Cruz, Santa Cruz, California, United States of America; 3 Division of Biological Sciences, Neurobiology Section, Howard Hughes Medical Institute, University of California San Diego, La Jolla, California, United States of America; University of Hong Kong, Hong Kong

## Abstract

**Background:**

The serine/threonine kinase BUB1 (Budding Uninhibited by Benzimidazole 1) was originally identified in yeast as a checkpoint protein, based on its mutant's incapacity of delaying the cell cycle in response to loss of microtubules. Our understanding of its function is primarily from studies carried out in yeast *S. cerevisiae*. It has been shown that it is a component of the mitotic spindle checkpoint and regulates the separation of sister chromatids through its downstream molecules. However, its roles in multi-cellular organisms remain unclear.

**Methods and Findings:**

In nematode *C. elegans*, rapid cell divisions primarily occur in embryos and in germline of postembryonic larvae and adults. In addition, a select set of cells undergo a few rounds of cell division postembryonically. One common phenotype associated with impaired cell division is described as Stu (Sterile and Uncoordinated) [1], [2]. We conducted a genetic screen for zygotic mutants that displayed Stu phenotype in *C. elegans*. We isolated seven Stu mutants that fell into five complementation groups. We report here that two mutations, *FanWang5* (*fw5*) and *FanWang8* (*fw8*) affect the *bub-1* gene, a homolog of yeast *BUB1*. Both mutant alleles of *fw5* and *fw8* exhibited variable behavioral defects, including developmental arrest, uncoordination and sterility. The number of postembryonically born neurons in the ventral cord decreased and their axon morphology was abnormal. Also, the decrease of neurons in the ventral cord phenotype could not be suppressed by a caspase-3 loss-of-function mutant. In addition, *bub-1*(*fw5* and *fw8*) mutants showed widespread effects on postembryonic development in many cell lineages. We found that *bub-1* functioned maternally in several developmental lineages at the embryonic stage in *C. elegans*. Studies in yeast have shown that BUB1 functions as a spindle checkpoint protein by regulating the anaphase promoting complex/cyclosome (APC/C). We performed double mutant analysis and observed that *bub-1* genetically interacted with several downstream genes, including *fzy-1*/*CDC20*, *mat-2*/*APC1* and *emb-27*/*APC6*.

**Conclusions:**

Our results demonstrate a conserved role of *bub-1* in cell-cycle regulation and reveal that *C. elegans bub-1* is required both maternally and zygotically. Further, our genetic analysis is consistent with that the function of *bub-1* in *C. elegans* is likely similar to its yeast and mammalian homologs.

## Introduction

Precise chromosome segregation during cell division is controlled by a feedback mechanism [3]. During the mitotic cell cycle, the metaphase-to-anaphase transition occurs after all chromosomes have established precise bipolar attachments to the mitotic spindles [4]. The spindle checkpoint inhibits anaphase onset until kinetochores are properly bound with the spindle microtubules [5], [6]. Malfunction of the spindle checkpoint leads to precocious anaphase and chromosomal missegregation, and results in subsequent loss of genetic fidelity. Misregulation of the spindle checkpoint has been suggested as a major cause of fatality and cancer [7], [8], [9], [10], [11]. In 1990s, several groups have isolated a number of genes involved in the budding yeast spindle checkpoint, including *MAD1* (Mitotic Arrest Deficient 1), *MAD2*, *MAD3* [12], *BUB1*, *BUB2*, *BUB3* [13], and *MPS1* (Monopolarspindle 1) [14]. BUB1 is a serine/threonine kinase that regulates the separation of sister chromatids. Studies from yeast have also shown that BUB1 acts through APC/C, a large multi-subunit E3 ubiquitin ligase [15], [16]. In addition, BUB1 localizes at the kinetochore during the very early stages of mitosis, and is required for kinetochore localization of MAD1 and MAD2, independent of its kinase activity [9]. Following the localization of BUB1, MAD1 then lowers the energy barrier of MAD2 and triggers MAD2 conformational change, allowing MAD2 binding to the APC/C activator CDC20. After the formation of the mitotic checkpoint complex (MCC), which contains BUBR1-BUB3-MAD2-CDC20, APC/C is inhibited by the complex [17]. This process results in the stabilization of securin, an inhibitor keeping separase inactive, and also hindrance of sister chromatids separation [18]. In mammalian cells, phosphorylation of CDC20 by BUB1 has also been shown to inhibit the function of CDC20 [19]. In *C. elegans*, components of the spindle checkpoint are functionally conserved [20], [21].

*C. elegans* has a single homolog of *BUB1*, *bub-1*. Antibody staining at one-cell stage shows that BUB-1 is an essential component in the mitotic kinetochore [22], consistent with its function in spindle checkpoint. RNAi of *bub-1* in wild type results in embryonic arrest, and partially restores mitotic timing at one-cell stage in conditional embryonic-lethal *apo-5*(*or358ts*) mutant embryos with cytoskeletal abnormalities, suggesting that *bub-1* may be associated with spindle checkpoint at the early embryonic stage [23]. Studies of putative downstream genes of *bub-1*: *mdf-1*/*MAD1*, *mdf-2*/*MAD2*, *mdf-3*/*MAD3*, and *fzy-1*/*CDC20* have also shown that these genes function during spindle checkpoint process [3], [24]. In a genetic screen for zygotic mutants that are likely associated with cell cycle defects, we isolated two *bub-1* mutant alleles. Our analysis shows that *bub-1* functions in multiple cell lineages and plays essential roles in the development of *C. elegans*.

## Results

### New Stu mutant screen

In *C. elegans*, some of the cell cycle mutants show morphological and behavioral defects including Stu and Emb (Abnormal EMBryogenesis). Emb commonly leads to embryonic lethality, while Stu mutants are often associated with defects in the development of gonads (sterility) or neurons in the ventral nerve cord (uncoordination) [25], [26], [27]. Some Stu mutants survive through embryonic development, likely due to maternal deposit of normal gene products [25]. In an effort to identify new cell cycle related genes in *C. elegans*, we conducted a clonal screen for Stu mutants using a GFP marker *juIs76* [P*unc-25::GFP*] that visualizes the D-type ventral cord motor neurons, which include embryonically born DD neurons and postembryonically born VD neurons [28]. We isolated seven Stu mutants from 3500 haploid genomes. By linkage group mapping and complementation tests, we found that these mutants fell into five complementation groups, of which one was a *mcm-5* allele that we had reported previously [29]. Table 1 shows the remaining four mutant complementation groups and their phenotypes. All animals isolated showed uncoordination, larval arrest, sterility and vulva defects (either vulvaless or protruding vulva). These phenotypic defects are commonly observed in animals with abnormal postembryonic development [27].

**Table 1:** Summary of the Developmental Phenotypes of Stu Mutants.

Mutation (genetic position)	Larval Arrest or Sterile %* (n**)				Vulval Morphology*** % (n)	
	L1/L2	L2/L3	L3/L4	Sterile adults	Pvl	Vul
N2	0	0	0	0 (264)	0	0 (234)
*fw2* (**V**:3.27∼3.89)	0	3.8	16.6	79.6 (320)	25.7	74.3 (113)
*fw3* (**V:** 3.27∼3.89)	0	2.4	8.6	89.0 (255)	13.7	86.3 (168)
*bub-1*(*fw5*) (**I:** 1.86)	9.0	16.7	31.8	42.5 (233)	4.3	95.7 (93)
*bub-1*(*fw8*) (**I:** 1.86)	9.1	8.7	56.2	26.0 (219)	8.0	92.0 (25)
*fw6* (**II:** 7.53∼13.65)	79.2	9.4	3.0	8 (371)	28.8	71.2 (73)
*fw9* (**II:** 11.99∼15.89)	0	9.0	27.6	63.3 (221)	49.1	50.9 (226)
*tm2815*/*tm2815*	4.9	12.3	22.2	60.5 (81)	4.9	95.1 (81)
*fw5*/*tm2815*	11.3	11.3	24.5	52.8 (106)	4.7	95.3 (106)
*fw8*/*tm2815*	9.7	15.9	21.2	53.1 (113)	8.0	92.0 (113)

* The percentage of each phenotype.

** Total number of examined animals.

*** For the sterile adult, vulva morphology was examined (see Materials and Methods).

Pvl: protruding vulva; Vul: vulvaless.

### All new Stu animals have motor neuron defects

**Figure 1:**
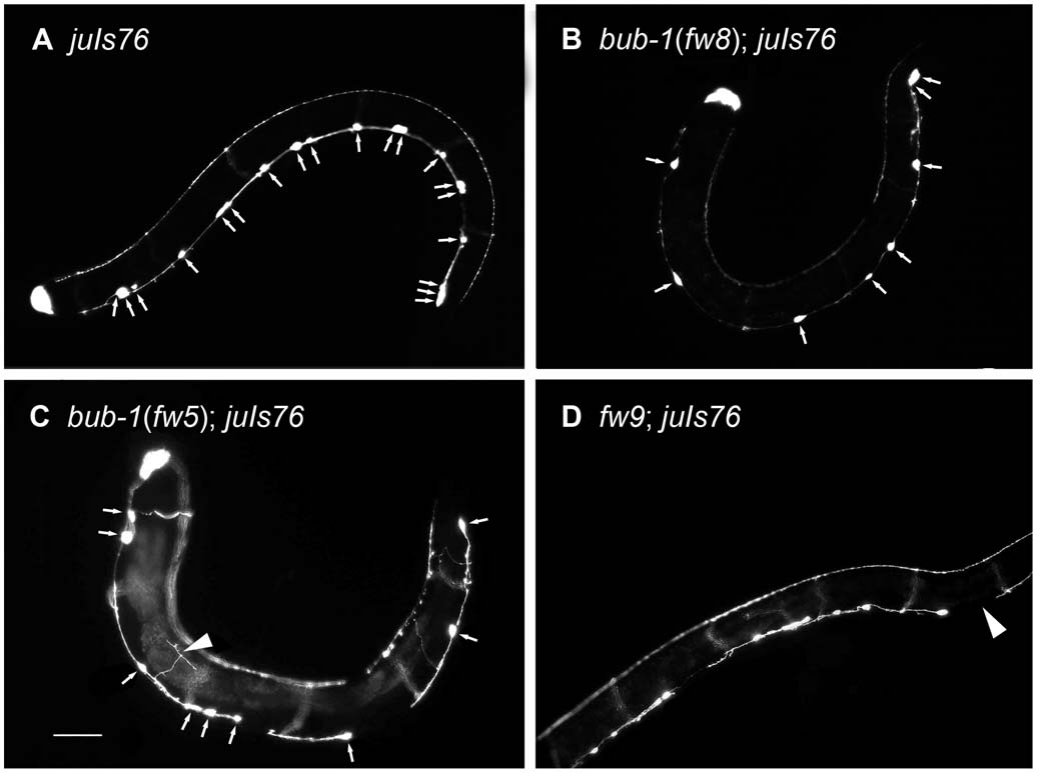
D-type Neuron and Axon Defects of Stu Mutants. (A) Wild-type animal (*juIs76*) has normal ventral cord D-type neurons. (B) *bub-1*(*fw8*) (C) *bub-1*(*fw5*) (D) *fw9*. Arrows show D-type neurons of the ventral nerve cord. In the *fw5, fw8*, and *fw9* mutants, the numbers of D-type neurons were decreased when compared to the wild-type animal. The arrowhead shows axon guidance defects in the Stu mutants. Anterior is to the left and ventral is down. The scale bar represents 50 µm.

The generation of adult ventral nerve cord involves a series of postembryonic cell division in late L1 larvae, resulting in a fixed number of neurons arranged in a stereotypic manner [30]. To evaluate the mutant phenotype, we counted the number of ventral cord motor neurons. In wild type animals, P*unc-25::GFP* visualizes 6 DD and 13 VD neurons in the ventral nerve cord [28]. The DD neurons are born at the embryonic stage, whereas VD neurons are born at the L1 larvae stage. All mutants had normal number of DD neurons in L1 larvae (data not shown). However, in later larvae (L2 or older) and adults, all mutants showed a general decrease in the number of GFP-expressing VD neurons (Figure 1). To confirm our findings, we used a pan-neuronal marker *evIs111* [31] and DAPI staining. The result showed that the mutants were missing many neurons, consistent with previous findings (data not shown). As reported previously, impairment in cell cycle often causes defects in cell morphology [26]. By examining the morphology of motor neurons in the mutants, we found that some VD neuron axons showed defective morphology in several mutants (Figure 1C and Table 2).

**Table 2:** Summary of the D-Type Motor Neuron and Axon Phenotypes of Stu Mutants.

Mutation	Number of D-type Neurons			Axon Defects**			
	L1 (n*)	L2 or Older Animals (n)	Range	L1 (n)	L2 or Older Animals (n)		
					Circumferential Guidance Defects	L-R Defects	Longitudinal Extension Defects
N2	6 (43)	19 (37)	18∼19	0 (51)	0 (59)	2 (59)	0 (59)
*tm2815*	6 (33)	12.4±2.0 (52)	9∼18	0 (33)	14 (52)	8 (52)	7 (52)
*fw2*	6 (100)	10.8±2.1 (262)	6∼17	0 (100)	43 (262)	40 (262)	N/A***
*fw3*	6 (100)	11.3±1.9 (239)	6∼17	0 (100)	41 (239)	20 (239)	N/A
*bub-1*(*fw5*)	6 (100)	8.7±1.7 (224)	6∼16	0 (100)	19 (224)	15 (224)	32 (224)
*bub-1*(*fw8*)	6 (111)	8.7±1.7 (244)	6∼15	0 (111)	52 (244)	14 (244)	12 (244)
*fw6*	N/A	11.4±2.4 (79)	7∼17	N/A	31 (79)	16 (79)	4 (79)
*fw9*	6 (100)	9.5±1.6 (216)	6∼14	0 (100)	83 (216)	56 (216)	27 (216)

* The number in the bracket is the total mutant number examined.

** Circumferential defects include premature stop or inappropriate branching. L-R defect refers to the D-type neuron commissures that extend from left side of the animals. Longitudinal extension defects were scored as regions that lack GFP-labeled axons.

*** N/A: not available.

### Both *fw5* and *fw8* are mutations in *bub-1*

To identify the corresponding genes of the new Stu mutations, we performed snip-SNP mapping (see Materials and Methods) [32]. We mapped *fw5* and *fw8* to the same interval (between the SNP marker of B0041:6882 and VF39H2L: 3079) on the chromosome I. Further, *fw5* and *fw8* failed to complement. Both *fw5* and *fw8* were balanced by *dpy-5*(*e61*) *unc-29*(*e403*) for stock keeping.

**Figure 2:**
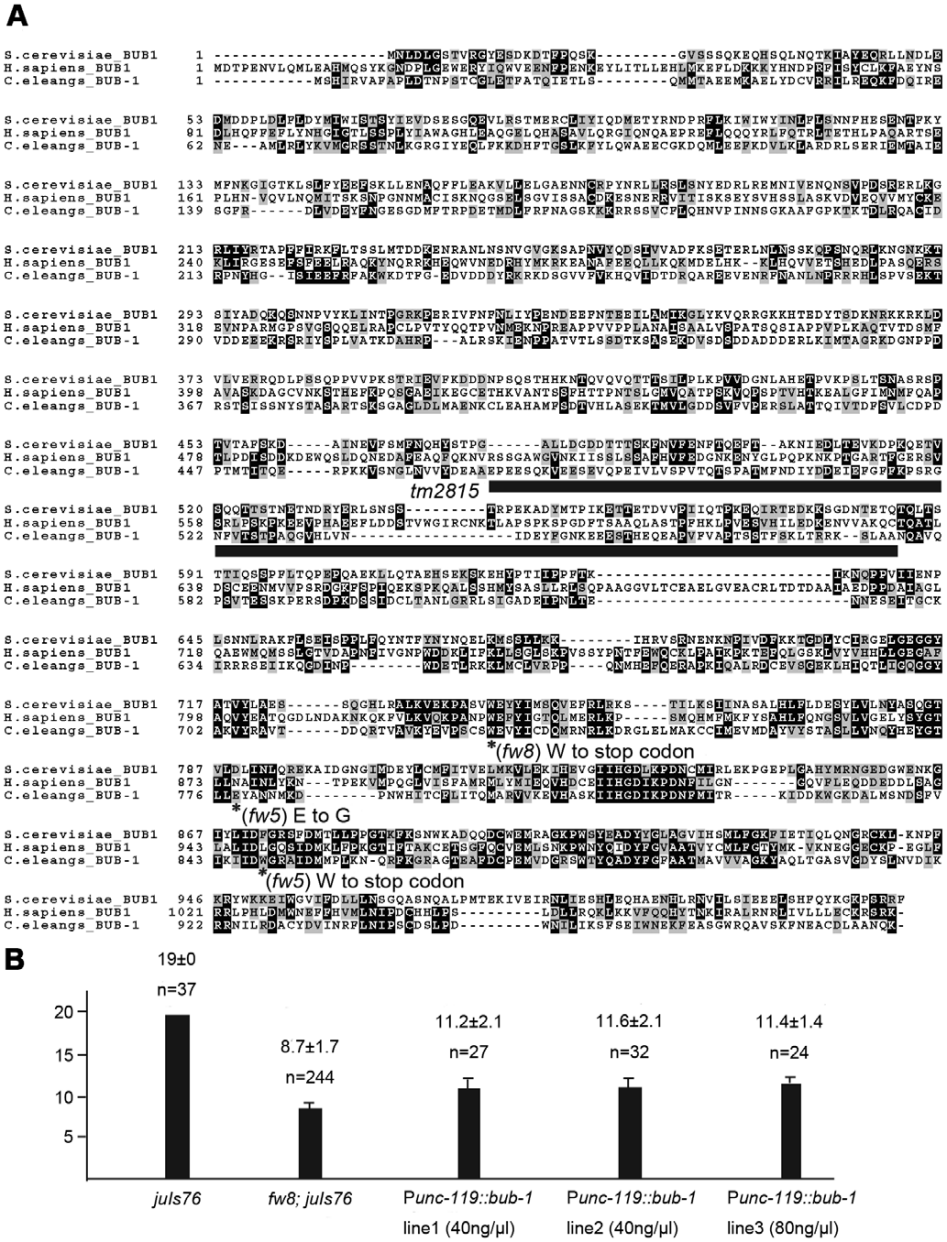
Sequence Comparison of BUB-1 and Rescue of *fw8*. (A) Alignment of *C. elegans* BUB-1 (http://www.wormbase.org/db/seq/protein?name=WP3ACE06251class=Protein), *S. cerevisiae* BUB1 (http://db.yeastgenome.org/cgi-bin/protein/protein?sgdid=S000003 420), and *H. sapiens* BUB1A (http://www.ensembl.org/Homo_sapiens/protview? peptide =  ENSP00000 302530). The conserved protein kinase domain of *C. elegans* BUB-1 is 29% identical with *S. cerevisiae* BUB1, and 31% identical with *H. sapiens* BUB1A. The protein sequences were obtained from wormbase, Ensembl, and SGD. BLASTS of two sequences were done using NCBI BLASTP. Multiple sequence alignment was done using ClustalW on the EMBL-EBI website (http://www.ebi.ac.uk/clustalw/index.html), and the shade was added by using BOXSHADE 3.21 (http://www.ch.embnet.org/software/BOX_form.html). The shade shows the conserved protein sequence. The black line indicates the deletion region of *tm2815*. (B) P*unc-119::bub-1* partially rescued the reduced D-type neuron defect of *fw8*. Y axis shows the D-type neuron numbers. The bars represent standard deviation (the t test compared to control *fw8*; *juIs76*: P<0.001).

We tested a set of RNAi clones covering the interval, and found that RNAi escapers of *bub-1* led to reduced number of D-type neurons as well as Emb (data not shown). We then sequenced *fw5* and *fw8*, and identified nucleotide alterations in the *bub-1* gene in both alleles (Figure 2A). In *C. elegans*, the *bub-1* gene encodes a 987aa protein with a conserved kinase domain at its C-terminal (Figure 2A). The mutations in *fw5* and *fw8* result in stop codon at W848 and W726, respectively, which produce truncated proteins lacking the kinase domain. We also obtained a deletion mutant, *tm2815*, which had an in-frame deletion of 105 amino acids from E473 to A586 in the middle of the protein, with unaffected kinase domain (Figure 2A). Homozygous *tm2815* animals displayed embryonic arrest, larval arrest and sterility. However, the phenotypes observed in the deletion allele were weaker than those of *fw5* or *fw8*. We also generated *tm2815*/*fw5* and *tm2815*/*fw8* animals and found that a larger number of surviving adult stage animals compared to homozygous *fw5* or *fw8* (Table 1). This result indicates that *tm2815* mutant behaves as a partial loss of function mutation, and *fw5* and *fw8* are more likely to be null mutations of *bub-1*.

We also performed transgenic rescue of the *bub-1* mutant using a PCR product which encompasses the region from 1.40-kb upstream to 0.82-kb downstream of the *bub-1* locus. We obtained two transgenic *fw8* homozygous lines after injecting the PCR product to the *dpy-5*(*e61*) *unc-29*(*e403*)/*fw8* animals. In both lines, the mutant phenotypes were fully rescued. Furthermore, expression of *bub-1* driven by a pan-neuronal promoter (the promoter of *unc-119*) was also able to rescue the neuronal defect of *fw8* as well. All three transgenic lines showed partial rescue of the loss of D type neurons (the t-test compared to control *fw8*; *juIs76*: P<0.001) (Figure 2B). These results suggest that BUB-1 is responsible for the Stu phenotypes of *fw8*, and *bub-1* functions in the nervous system in a cell-autonomous manner.

### The neuronal defect of *fw8* is unlikely caused by caspase-dependent programmed cell death

As mentioned earlier, the number of VD neurons was reduced in *bub-1*(*fw8*) mutants (Figure 1B). The loss of neurons could be due to abnormal cell division [33] or enhanced apoptosis [34], [35]. To examine these possibilities, we constructed a *bub-1*(*fw8*); *ced-3*(*n717*) double mutant in which programmed cell death would be blocked due to the loss of CED-3 caspase activity [36]. We found that *bub-1*(*fw8*) and *bub-1*(*fw8*); *ced-3*(*n717*) resulted in similar numbers of D-type neurons [average 8.7 (n = 244) and 8.3 (n = 31), respectively] (Table 3). This result indicates that caspase-dependent apoptotic cell death is unlikely responsible for the loss of motor neuron in *bub-1*(*fw8*). However, we could not rule out the possibility of caspase-3-independent cell death in *bub-1*(*fw8*) mutant.

**Table 3:** Number of D Type Motor Neuron in *bub-1*(*fw8*); *ced-3*(*n717*) Mutants.

Mutation	Number of D-type Neurons
*bub-1*(*fw8*)	8.7±1.7 (244)
*bub-1*(*fw8*); *ced-3*(*n717*)	8.3±2.2 (31)

* The number in the bracket is the total mutant number examined.

### *bub-1* is required both maternally and zygotically

**Figure 3:**
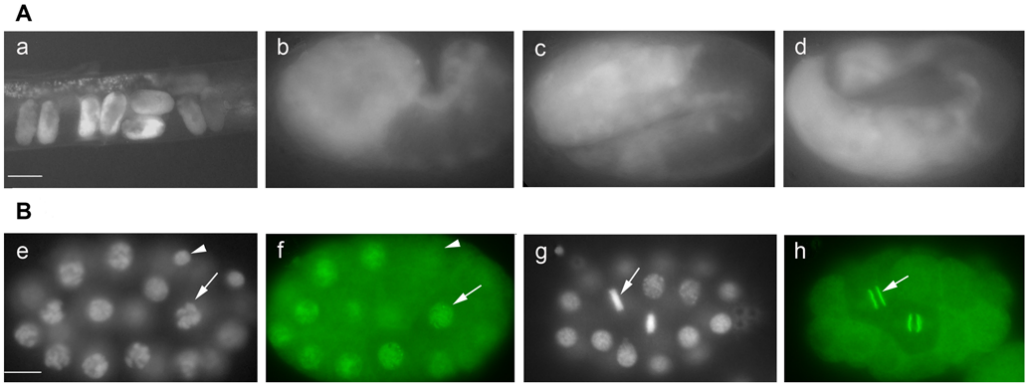
Expression Pattern of *bub-1*. (A) P*bub-1::GFP* was widely expressed in the embryonic stage: (a) early *C. elegans* embryos in the gonad of an adult animal; (b) comma stage embryo; (c) two-fold stage embryo; and (d) three-fold stage embryo. The scale bar represents 50 µm for (a) and 10 µm for (b), (c), and (d). (B) Antibody staining of BUB-1 in embryo nucleus. (e) DAPI staining and (f) antibody staining of BUB-1 of the same embryo. (g) DAPI staining and (h) antibody staining of BUB-1 of the same embryo. In (e) and (f), some of the cells (as shown by the arrow) were at the prophase of mitosis and BUB-1 was localized in the nuclei. The arrowheads show the non-dividing cell where BUB-1 was not expressed. In (g) and (h), one cell (as shown by the arrow) was at the metaphase of mitosis and BUB-1 was localized to the kinetochore. The scale bar represents 10 µm.

The fact that *bub-1* mutants caused only postembryonic-born VD neuron defects suggested two possible reasons: 1) *bub-1* is a maternal gene and 2) *bub-1* is specifically required at postembryonic stages. A *bub-1* promoter driven GFP was widely expressed from early embryonic stages to three fold stage (Figure 3A). Anti-BUB-1 antibody staining also showed BUB-1 was present at the one-cell stage [22], and during the late embryonic stage (Figure 3B). To examine the roles of *bub-1* in early embryos, we fed *bub-1* RNAi to the *eri-1*(*mg366*); *juIs76* animals, which sensitized the RNAi effect [37]. We found that approximately 90% of the progenies from the RNAi-fed parents showed the Emb phenotype (n = 779). To characterize at which stage the embryos arrested, we stained the Emb embryos with DAPI and found that approximately 1% of them arrested at the early embryonic stage (an average of twenty nuclei, n = 107), while about 94% arrested at late embryonic stage (an average of 100 nuclei, n = 107). Only a few of the embryos arrested at the comma stage (5.6%, n = 107). These observations indicate that BUB-1 is required maternally during embryogenesis, in addition to its zygotic roles in postembryonic development.

### Effects of *bub-1* in postembryonic development

In *C. elegans*, multiple types of tissues undergo several rounds of cell divisions during postembryonic development. Using a panel of markers, we examined the development of several tissues in *bub-1* mutants as described below.

#### Intestinal nuclei division but not endoreduplication was defective

**Figure 4:**
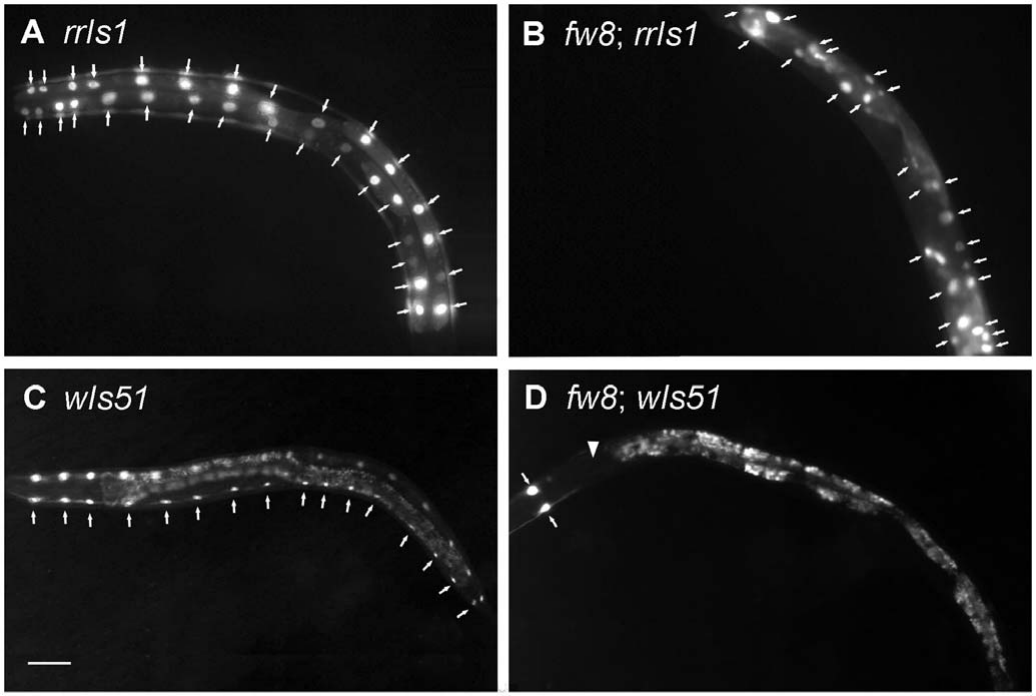
The Intestine Nucleus and Seam Cell Number Decreased in *fw8* Mutants. (A) The *rrIs1* [P*elt-2::GFP*] marker visualizes the nuclei of the intestine cells. (B) *fw8* animals exhibited a decrease in the intestine nuclei number, as shown by the arrowhead. The arrow indicates intestine nuclei. (C) *wIs51* [*SCM::GFP, unc-119*(*+*)] animal exhibits two rows of seam cells, and each row has sixteen seam cells indicated by the arrow. (D) *fw8* exhibited a decrease of seam cells. Only two seam cells at the head region were observed, and most of the seam cells were missing, as shown by the arrowhead. Anterior is to the left. The scale bar represents 50 µm.

**Figure 5:**
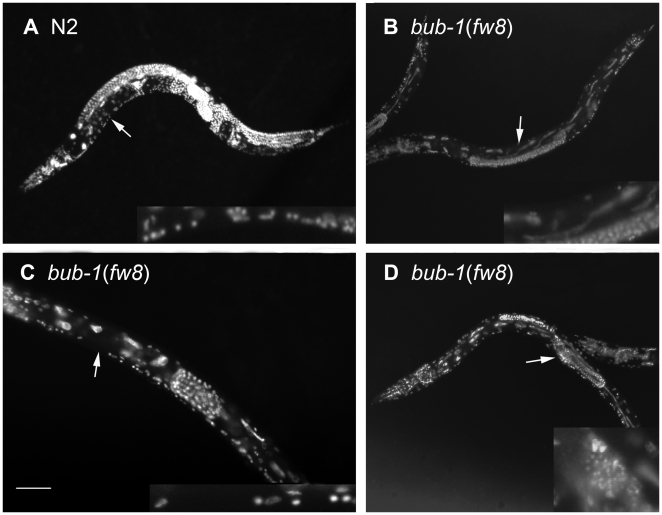
DAPI Staining Images of *bub-1* Mutants. (A) wild-type animal N2. (B) The arrow indicates the elongated intestine cell in *bub-1*(*fw8*) animal. (C) Compared with N2 animals, the ventral cord of *bub-1*(*fw8*) had fewer neuron numbers (shown by the insert). (D) The arrow indicates the sperm in the *bub-1*(*fw8*) mutant. The bar represents 100 µm.

**Figure 6:**
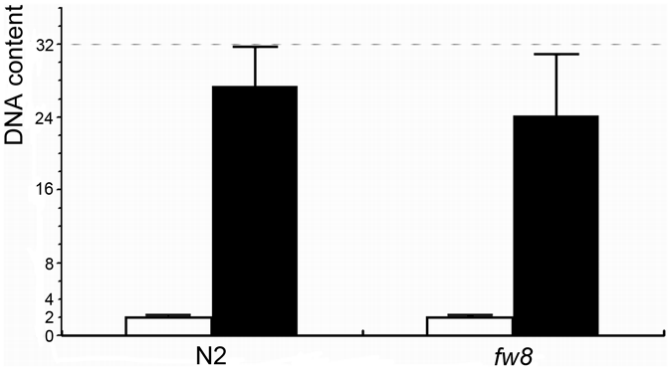
Intestinal Ploidy Measurement of *bub-1* Mutants. Body wall muscle nuclei were used as an internal 2 n standard. White bar indicates the average DNA content±s.d. of 10 body wall muscle nuclei in three independent animals. Black bar indicates the average DNA content±s.d of 30 intestinal nuclei in three independent animals.

The transgenic GFP line *rrIs1* was used to visualize the nuclei of the intestine cells (Figure 4A). In wide type late L1 animals, the intestine cells have 30 to 34 diploid nuclei. All intestinal nuclei endoreduplicate their DNA prior to each of the four molts, thereby producing the 32 n DNA content nuclei in the adult intestine [39]. We found, however, about 24 intestinal nuclei in the *bub-1*(*fw8*) mutant L4 larvae (n = 17) (Figure 4B). In addition, some of the intestinal nuclei were elongated and showed a thread structure, suggesting a defect in chromosomal segregation [25]. This observation was consistent with the DAPI staining experiment (Figure 5B). Furthermore, we checked the DNA content of the intestinal nuclei in the *bub-1*(*fw8*) L4 or adults. Using body wall muscle nuclei as an internal 2 n control, we determined that the amount of DNA in the intestinal lineages was 24.3 n in the *bub-1*(*fw8*) mutant, while 28.4 n in the WT (Figure 6). If the arrest of cell division prior to L4 stage and the lack of the last DNA replication before L4 to adult molt are taken into account, we tend to believe that the intestinal nuclei endoreduplication might not be affected by the loss of *bub-1* function. However, the cell division may be affected.

#### Division of seam cells was severely disrupted

We used a transgenic GFP line *wIs51* to visualize the nuclei of seam cells [40] (Figure 4C). Ten seam cells aligned on each side of the body undergo stage-specific division patterns at each of four (L1–L4) postembryonic larval stages. From the L2 to L4 stage, the wild type animal has 16 seam cells [38]. In most of the *bub-1*(*fw8*) L4 mutants, only the two most anterior seam cells H0 were present (Figure 4D). These H0 cells normally do not undergo postembryonic division [41]. These results indicate a severe failure in postembryonic division of seam cells.

#### Gonad development was severely impaired

**Figure 7:**
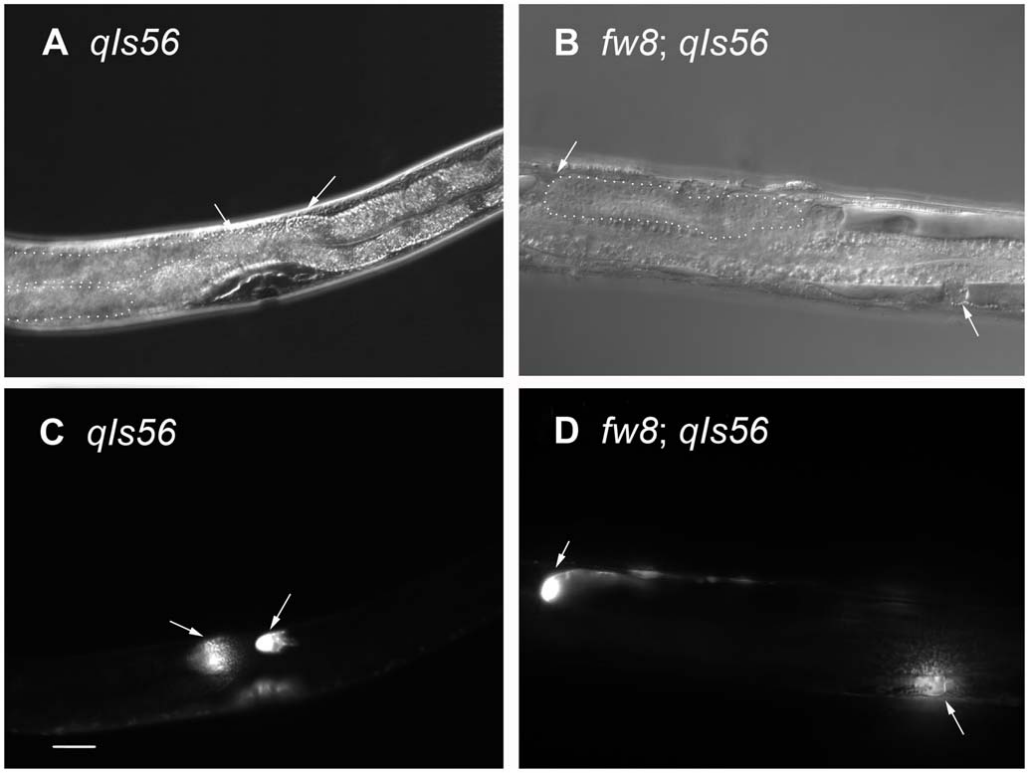
Nomarski and DTC Marked GFP (*qIs56*) Images of *bub-1* Mutants. (A) The DIC and (C) the DTC GFP picture of the same N2. (B) The DIC and (D) the DTC GFP picture of the same *bub-1*(*fw8*) mutant. The DTC GFP cells showed that the *bub-1*(*fw8*) mutant gonad arm could not grow to form the U shape gonad. Anterior is to the left and ventral side is down. One gonad in each animal in (A) and (B) was outlined in a dotted line. Dorsal is up in 7a and 7c; and dorsal is facing out the paper in 7b and 7d. The scale bar represents 25 µm.

The transgenic GFP line *qIs56* allowed us to visualize the two distal tip cells (DTCs) of the U-shaped gonads [42] (Figure 7A and 7C). The gonad arms acquire their U-shape by directed migration of the DTC. The arm elongation begins at the L2 stage and continues until the L4 molt [38]. We observed that about half of *bub-1*(*fw8*) animals showed only one gonad arm, and most of them stopped development prematurely (n = 32) (Figure 7B and 7D). Among the 48 gonad arms scored, 9 grew one quarter or less of the normal gonad length; 15 gonad arms grew less than one half of the normal length; and 10 gonad arms grew about three quarters of the normal length. Furthermore, the number of germ cells in *bub-1*(*fw8*) was decreased to about 117 per arm (n = 11) (compared to about 1000 in wild type). In the abnormal gonads, we did not observe any eggs. Sperms, however, formed only in 2 of the 9 *bub-1* mutant animals observed by DAPI staining (Figure 5D).

#### Ventral cord motor neurons

**Figure 8:**
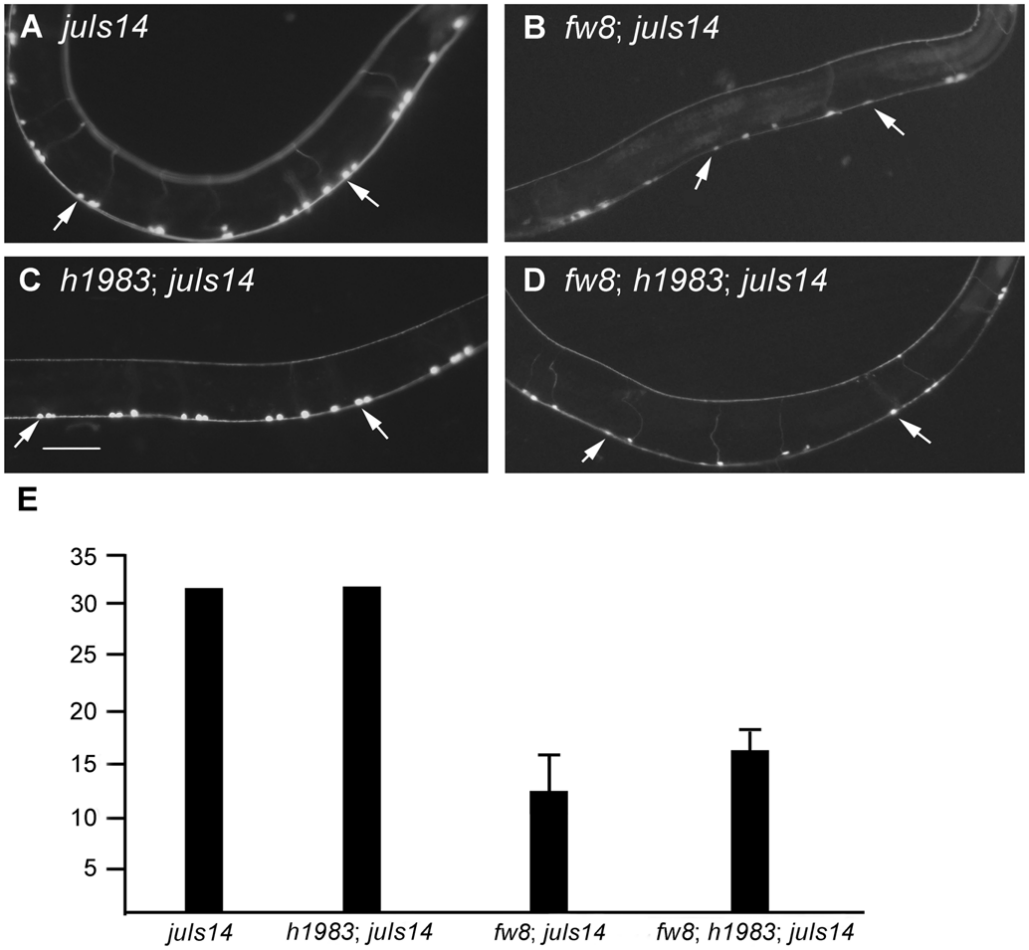
Weak Allele of *fzy-1*(*h1983*) Suppressed Neuron Decrease Phenotype of *bub-1*(*fw8*). (A) *juIs14* [P*acr-2::GFP*] strain visualizes A and B-type neurons, (B) *bub-1*(*fw8*) had less A and B type neuron. (C) *fzy-1*(*h1983*) did not affect A and B-type neuron fate. (D) *fzy-1*(*h1983*) could partially suppress *bub-1*(*fw8*) in neuron counting number. Arrows indicate some of the A and B-type neurons. The bar represents 50 µm. (E)Y axis shows the A and B-type neuron numbers (not including neurons in the head ganglia). Error bars represent standard deviation (the t-test compared to control *fw8*; *juIs14*: P<0.001).

We used a transgenic GFP strain, *juIs14* [33], to visualize the cholinergic DA, DB, VA, and VB neurons (Figure 8A). We observed a decreased number of neurons expressing GFP in the *bub-1*(*fw8*) mutant. Normally, embryonic-born DAs and DBs have commissural projections to the dorsal cord, while postembryonic-born VAs and VBs do not [38]. We found that the number of commissural projections to the dorsal cord was unchanged in the *bub-1*(*fw8*) mutant, and the axons of these neurons did not show any morphological defects (data not shown). Therefore, embryonic-born DAs and DBs were not affected, while most postembryonic-born VAs and VBs were missing in the *bub-1*(*fw8*) mutant.

### Genetic interaction analysis supports a role of BUB-1 in the spindle checkpoint pathway

Previous studies have shown that several components of the spindle assembly pathway are functionally conserved in nematodes and yeast [20], [21]. For example, the loss-of-function of *mdf-1*/*MAD1* causes embryonic and larval arrest [3], similar to the yeast mutant. Further, the lethal phenotype of *mdf-1*/*MAD1* is suppressed by the mutations in the downstream genes, such as *fzy-1*/*CDC20* [20], and APC/C homologues, such as *emb-30*/*APC4* [43] and *such-1*/*APC5-like* [44]. To test if *bub-1* acts in the spindle checkpoint pathway, we examined genetic interactions between *bub-1*(*fw8*) and several candidate downstream genes.

*fzy-1*/*CDC20* is an activator of APC/C at the transition from metaphase to anaphase. A previous study demonstrated that BUB1 inhibited CDC20 in cultured mammalian cells [19]. In *C. elegans*, *fzy-1* (*h1983*) did not exhibit major developmental abnormalities, except for the smaller brood size [20]. Consistently, we found that *fzy-1*(*h1983*) did not affect postembryonic neuronal cell division (Figure 8C). In the wild type worm, about 33 DA, DB, VA, and VB neurons are present along the ventral cord, not including the head ganglia neurons. In *bub-1*(*fw8*) worm (n = 105), only 13 were present. However, in the *fzy-1*(*h1983*); *bub-1*(*fw8*) double mutant, there were approximately 17 DA, DB, VA, and VB neurons present (n = 26). Moreover, 51.06% of double mutants of *fzy-1*(*h1983*); *bub-1*(*fw8*) survived to adulthood, compared to 26.03% of *bub-1*(*fw8*) (Figure 8E). These results indicate that the effects from the *bub-1* mutation are partially suppressed by the mutation of *fzy-1*/*CDC20*, consistent with *fzy-1* acting downstream of *bub-1*.

*fzr-1*/*CDH1*/*HCT1* is another activator of APC/C required for exit of mitosis [45] and shows sequence similarity to *fzy-1*. In *C. elegans*, *fzr-1* (*ok380* and *ku298* alleles) did not exhibit major developmental abnormalities. To examine the genetic interaction between *bub-1* and *fzr-1*, we made double mutants of *bub-1*(*fw8*) and *fzr-1* (*ok380* and *ku298* alleles). The survivability of both allelic double mutants was indistinguishable from *bub-1*(*fw8*) (data not shown). Thus, *fzy-1* is most likely a downstream regulator of *bub-1*, but not *fzr-1*, in *C. elegans*.

*mat-2*/*APC1* and *emb-27*/*APC6* are two *APC*/*C* subunits. Previous studies have shown that these subunits might function during meiosis. *mat-2*(*ax102*) and *emb-27*(*g48*) are temperature sensitive mutants that can be maintained as fertile adults at 15°C. By temperature shift experiments (see Methods), we observed that, while most *bub-1*(*fw8*) mutants arrested at different larvae stage, 75.5% and 69.8% of *bub-1*(*fw8*); *mat-2*(*ax102*) and the *bub-1*(*fw8*); *emb-27*(*g48*) double mutants respectively developed into sterile adults (Table 4). This study showed that *mat-2*(*ax102*) and *emb-27*(*g48*) partially suppressed the larval arrest phenotype of *bub-1*(*fw8*). It suggests that *bub-1* may function through the downstream factors of APC/C.

**Table 4:** Adult Sterility in Double Mutants of *bub-1*(*fw8*) with *APC*/*C* Subunits.

Mutation	Adult Sterility
*bub-1*(*fw8*)	26.0% (219)
*bub-1*(*fw8*); *mat-2*(*ax102*)	75.5% (73)
*bub-1*(*fw8*); *emb-27*(*g48*)	69.8% (69)

* The number in the bracket is the total mutant number examined.

## Discussion

### Identification and characterization of loss-of-function mutations of *C. elegans bub-1*, a cell cycle spindle checkpoint gene

Our conclusion that *fw5* and *fw8* are loss of function mutations in *bub-1* is based on the following results: 1) they failed to complement with each other; and were mapped to the same genetic interval; 2) RNAi against *bub-1* exhibited the same phenotypes as *fw5* and *fw8*; 3) sequencing data showed that *fw5* and *fw8* both contained nonsense mutations in the *bub-1* coding sequence; 4) an in-frame deletion mutant of *bub-1* (*tm2815*) failed to complement with *fw5* and *fw8*, and exhibited weaker phenotypes than *fw5* and *fw8*; and 5) *fw5* and *fw8* could be rescued by *bub-1* DNA and partially rescued by expression of *bub-1* gene driven by a pan-neuronal promoter.

### BUB-1 may have both kinase-dependent and kinase-independent functions

Compared to our *bub-1* mutant *fw5* and *fw8*, the deletion mutant *bub-1*(*tm2815*) showed milder defects. This is likely due to an existing partial function of *bub-1*(*tm2815*). Based on sequence alignment among different species, Bub1 has a conserved kinase domain at the C-terminus. Both *fw5* and *fw8* have premature stop codon prior to the kinase domain, whereas *bub-1*(*tm2815*) has an in-frame deletion which leaves an intact kinase domain. This might explain why *fw5* and *fw8* have more severe defects than *bub-1*(*tm2815*). Furthermore, this difference might suggest that *bub-1* functions beyond a kinase. In yeast, BUB1 is required for kinetochore localization of MAD1 and MAD2 independent of its kinase activity [9]. Further, *mdf-1* (mitotic arrest defective) and *mdf-2* were identified as homologs of *MAD1* and *MAD2*, and both exhibited conserved function in nematode and yeast [3]. Whether or not the kinase-independent function of *bub-1* exists in *C. elegans* still needs to be investigated further.

Our studies demonstrate that the cell cycle control gene *bub-1* functions widely in the development of *C. elegans*. The *bub-1* null mutants exhibited defects in several developmental lineages, including seam cells, intestine nuclei, vulva, gonad, germ cells, and ventral cord neurons. Other postembryonic cell lineages we inspected were also defective in *bub-1* mutants (data not shown). In *bub-1*(*fw5, fw8*) mutants, all of the neurons in the ventral cord developed at the embryonic stage were intact, such as DAs, DBs, and DDs; while most of the postembryonic-born neurons were missing, such as VAs, VBs, and VDs. Our RNAi experiment shows that *bub-1* is a maternal gene and the maternal effect of *bub-1* is strong enough to support embryonic development even to the adult stage in *bub-1* mutants. In *C. elegans*, some cell cycle-related genes also show long lasting maternal function. For example, *cye-1* Cyclin E deletion animals showed surprisingly normal development until the L3 stage, although RNAi resulted in embryonic lethality at nearly the hundred-cell stage [46], [47].

### The endoreduplication may not be affected by the loss of *bub-1* function

Metazoans have various types of cell cycles during development. Endoreduplication is a specific type of cell cycle that skips the M phase. In *C. elegans*, such endoreduplication type of cell cycle takes place in the intestine and hypodermis during development [39]. Intestinal nuclei go through an endoreduplication cycle before each molt, which results in adults with intestinal nuclei with a 32 n DNA content. In adult animals or L4 with *bub-1*(*fw8*) mutants, we found that the amount of DNA was not affected. This result suggests that *bub-1* function is specifically required for the spindle checkpoint in the M phase, which is missing from the endoreduplication in the *C. elegans* intestinal cells.

### The *bub-1*-associated spindle checkpoint pathway is conserved in *C. elegans*

Studies in yeast and mammals show that BUB1 kinase acts on the upstream of CDC20 [17], [18], [19]. Consistent with these studies, we found that *h1983*, a partial loss of function allele of *fzy-1*/*CDC20*, partially suppressed the *bub-1*(*fw8*) phenotype. In *fzy-1*(*h1983*); *bub-1*(*fw8*) double mutant, the function of *bub-1* was abolished and *fzy-1* was not inhibited. As a result, the *fzy-1*(*h1983*) mutation partially complemented this defect and suppressed the phenotype of *bub-1*(*fw8*). *mat-2*(*ax102*) and *emb-27*(*g48*) also partially suppressed the *bub-1*(*fw8*) phenotype. These genetic analyses support the idea that *fzy-1* and APC/C are downstream targets of *bub-1* in *C. elegans*. However, we do not know whether BUB-1 functions through MDF-1/MAD1, MDF-2/MAD2 or phosphorylation of FZY-1 to inhibit FZY-1.

## Materials and Methods

### Culture conditions and strains

*C. elegans* strains were maintained at 20°C on nematode growth medium (NGM) seeded with *E. coli* strain OP50 as described by Brenner [48]. The temperature-sensitive strains were maintained at 15°C, and examined at 25°C. Mutations used in this study were as follows: LGI: *bub-1*(*tm2815*) LGII: *emb-27*(*g48*), *fzr-1*(*ok380*, *ku298*), *fzy-1*(*h1983*), *mat-2*(*ax102*) LGIV: *eri-1*(*mg366*), *ced-3*(*n717*). Transgenic markers were: *juIs76* [P*unc-25::GFP*] [28]; *oxIs12* [P*unc-47::GFP*] [49]; *juIs14* [P*acr-2::GFP*] [33]; *qIs56* [P*lag-2::GFP; unc-119*(*+*)] [42]; *rrIs1* [P*elt-2::GFP*] [50]; *wIs51* [*SCM::GFP*, *unc-119*(*+*)] (SCM stands for seam cell specific promoter), [40]; and *evIs111* [P*F25B3.3::GFP*] [51].

### Genetic screen for Stu mutants

CZ1200 *juIs76* [P*unc-25::GFP*] animals were synchronized by lysing the adult hermaphrodites, using alkaline hypochlorite (0.5% sodium hypochlorite, 0.5 N NaOH). The synchronized L4 animals were then treated with 50 mM ethyl methane sulfonate as described [52]. F1 progeny were placed on 1 animal per plate. Sterile or larval arrested, and Stu animals among the F2 progeny were examined further for the number and morphology of postembryonic neurons using the P*unc-25::GFP* marker. Strains were maintained by propagating heterozygous animals.

### Out-crossing, mapping and complementation testing

All of the mutants were out-crossed at least twice with N2. The mutants were mapped using standard snip-SNP assay [32] and the three-factor mapping technique [52]. The mutants mapped to similar genetic loci were tested. *fw2* and *fw3* were allelic, as were *fw5* and *fw8*. For the complementation procedure, we used heterozygous *bub-1*(*fw5*)/+ males to cross with the balanced strain *dpy-5*(*e61*) *unc-29*(*e403*)/*dpy-5*(*e61*) *bub-1*(*fw8*). The progenies *bub-1*(*fw5*)/*dpy-5*(*e61*) *bub-1*(*fw8*) were sterile and uncoordinated, which was similar to the *fw5* or *fw8* homozygous mutants. Complementation tests with known genes were also performed. These genes were within the same loci and generated similar phenotypes.

### Phenotypic quantification of Stu mutants

L4 Heterozygous balanced mutants, such as *dpy-5*(*e61*) *unc-29*(*e403*)/*bub-1*(*fw8*), were cultured at 20°C and transferred everyday to new plates to obtain synchronized progenies. From these plates, the uncoordinated F1 animals were transferred to new plates and cultured for about 5 days at 20°C to quantify the final phenotype. Larval arrest phenotype was quantified according to body size. The absence of fertilized eggs was scored as sterility. For the adult Stu animals, vulval morphology was quantified by mounting them in 2% agar pads and viewed under a stereoscope. Animals with protruding vulva were scored as Pvl, and others without vulva were scored as Vul. The D-type neuron phenotype of L1 stage animals were quantified 10 hours later after lysing the adult heterozygous mutants (+/−), using alkaline hypochlorite. A quarter of the population in these L1 animals become homozygous mutants (−/−).

### Nomarski fluorescent microscope examination

Live animals were mounted to M9 solution in 2% agar pads and viewed under Leica and Zeiss microscopes. Images were captured using a Leica DC500 or a Zeiss AxioCam.

### Molecular analysis of *bub-1*

To identify the mutations in *fw5* and *fw8*, the sequences for the exons and exon-intron boundaries of *bub-1* were amplified from homozygous mutant animals using the following primers: first pair (5′gcgtcctttctactttga3′, 5′gcttttcccgagttattt3′); second pair (5′ttcaatgcgggttctaag3′, 5′ctggagggttaccatctt3′); third pair (5′tcgtcggatacaaagtct3′, 5′ggttggagcaacaaatac3′); fourth pair (5′tttcaaaccgtctcgtgg3′, 5′tcaggcgattccgcattt3′); fifth pair (5′gtcaaggtggatacgctaa3′, 5′actttcctgcaacaacga3′); and sixth pair (5′aatggctgtcgttgttgc3′, 5′ ttctaccgtgatgggtct3′). The mutations were confirmed by sequencing from both directions (through two different reactions). To generate a *bub-1* promoter-driven GFP construct, duplex PCR [53] was conducted to amplify the 1266 bps *bub-1* upstream sequence from N2 genomic DNA using the following primer set. 5′gattcccacaagtaggtc3′ and 5′agtcgacctgcaggcatgcaagcttcaaagtagaaaggacgcga3′. The final P*bub-1::GFP* DNA fragment (100 ng/µl) was injected into the N2 strain using a pRF4 plasmid (100 ng/µl) as co-injection marker. Two lines were obtained and both showed similar expression patterns.

### Microinjection to rescue *fw8* phenotype

To rescue *bub-1*(*fw8*), a region from 1.40-kb upstream to 0.82-kb downstream of the *bub-1* locus was amplified from genome DNA with PCR primers 5′tcgaatcgcagttcttgtc3′ and 5′gagccatcagcttggttgt3′. The PCR product was injected (co-injected with pRF-4[*rol-6*(*su1006*)] at 80 ng/µl) to the balanced strain *dpy-5*(*e61*) *unc-29*(*e403*)/*bub-1*(*fw8*) at 40 ng/µl. In total, we obtained two transgenic lines. The full coding sequence of *bub-1* was cloned into the plasmid pBY103 (kindly provided by Dr. X. Huang) which contained the promoter of *unc-119* [54]. Based on their cloning data, *Kpn*I/*Sac*I double digestion was used to obtain the PCR product of *bub-1* genomic sequence. The P*unc-119::bub-1* plasmid was injected (co-injected with pRF-4[*rol-6*(*su1006*)] at 80 ng/µl) to the balanced strain *dpy-5*(*e61*) *unc-29*(*e403*)/*bub-1*(*fw8*) at 40 ng/µl. We obtained two transgenic lines. However, at 80 ng/µl, we obtained only one line and in the F1 progenies many larvae were lethal.

### Antibody staining

The freeze-crack method was used for permeabilization and fixation of the embryos [55]. The rabbit polyclonal antibody against BUB-1 (1∶1000, a gift from Dr. Hyman [22]) was used, followed by the FITC conjugated mouse anti-rabbit secondary antibody (1∶1000).

### RNAi by feeding

RNAi clones were made by J. Ahringer's laboratory [56], and obtained from the MRC service (UK). The bacteria expressing dsRNA of appropriate genes were cultured at 37°C overnight and seeded onto the NGM plates (containing 50 µg/mL Amp, 1 mM IPTG). The plates were kept at room temperature for two days. Three L4 CZ5547 (*mg366*; *juIs76*) animals were transferred to the plates. Two days later, the animals were then transferred to a second plate with the same interfering bacteria. About 10 hours later, the animals were removed and the embryos were cultured for a period of several days in order to examine the phenotype. The results were scored from the second plate, which displayed a better representation of the gene's mutant phenotype.

### DAPI staining

Approximately 30 mutant animals were placed into M9 on a microscope slide and covered with coverslip. The slide was quickly frozen in liquid nitrogen and put into a pre-cooled iron block. The coverslip was then quickly removed. The slide was sequentially placed in methanol and then acetone for 10 minutes each at −20°C. After air drying, animals were treated with 4′,6-diamidino-2-phenylindole dihydrochloride (DAPI) and covered with a coverslip [55].

### DNA quantitation

To quantitate DNA content, nuclei images of DAPI-stained animals were taken with a Zeiss AxioCam, and images were analyzed with NIH ImageJ 1.40 g software. Using body wall muscle nuclei as a 2 n DNA standard, C values of intestinal nuclei were estimated by their DAPI-based densitometric quantifications [57], [58].

### Double mutant analysis of *bub-1*(*fw8*) and *mat-2*(*ax102*), *emb-27*(*g48*)

Young adult stage double mutants *dpy-5*(*e61*) *unc-29*(*e403*)/*bub-1*(*fw8*); *mat-2*(*ax102*) and *dpy-5*(*e61*) *unc-29*(*e403*)/*bub-1*(*fw8*); *emb-27*(*g48*) were cultured at 15°C for two hours to lay eggs to bypass the meiosis requirement of APC/C. Then, the eggs were transferred to a temperature of 25°C. The phenotypes were scored as above. The *dpy-5*(*e61*) *unc-29*(*e403*)/*bub-1*(*fw8*) animals were treated with the same procedures as the control.
